# A Proteomic Approach to Elucidate the Changes in Saliva and Serum Proteins of Pigs with Septic and Non-Septic Inflammation

**DOI:** 10.3390/ijms23126738

**Published:** 2022-06-16

**Authors:** María José López-Martínez, José Joaquín Cerón, Alba Ortín-Bustillo, Damián Escribano, Josipa Kuleš, Anđelo Beletić, Ivana Rubić, Juan Carlos González-Sánchez, Vladimir Mrljak, Silvia Martínez-Subiela, Alberto Muñoz-Prieto

**Affiliations:** 1 Interdisciplinary Laboratory of Clinical Analysis of the University of Murcia (Interlab-UMU), Department of Animal Medicine and Surgery, Veterinary School, Regional Campus of International Excellence Mare Nostrum, University of Murcia, Espinardo, 30100 Murcia, Spain; mariajose.lopez28@um.es (M.J.L.-M.); alba.ortinb@um.es (A.O.-B.); det20165@um.es (D.E.); irubic@vef.unizg.hr (I.R.); silviams@um.es (S.M.-S.); 2 Clinic for Internal Diseases, Faculty of Veterinary Medicine, University of Zagreb, Heinzelova 55, 10000 Zagreb, Croatia; jkules@vef.unizg.hr (J.K.); abeletic@vef.hr (A.B.); vmrljak@vef.unizg.hr (V.M.); 3 BioQuant, Faculty of Biosciences, Heidelberg University, Im Neuenheimer Feld 267, 69117 Heidelberg, Germany; juan-carlos.gonzalez@bioquant.uni-heidelberg.de

**Keywords:** sepsis, proteomics, pigs, saliva, serum

## Abstract

Sepsis is a systemic inflammatory response triggered by an infectious agent and is recognized by the World Health Organization as a global concern, since it is one of the major causes of severe illness in humans and animals. The study of the changes that can occur in saliva and serum in sepsis can contribute to a better understanding of the pathophysiological mechanisms involved in the process and also to discover potential biomarkers that can help in its diagnosis and monitoring. The objective of this study was to characterize the changes that occur in the salivary and serum proteome of pigs with experimentally-induced sepsis. The study included five pigs with sepsis induced by LPS administration and five pigs with non-septic inflammation induced by turpentine for comparative purposes. In saliva, there were eighteen salivary proteins differentially expressed in the sepsis condition and nine in non-septic inflammation. Among these, significant increments in aldolase A and serpin B12 only occurred in the sepsis model. Changes in aldolase A were validated in a larger population of pigs with sepsis due to *Streptococcus suis* infection. In serum, there were 30 proteins differentially expressed in sepsis group and 26 proteins in the non-septic group, and most of the proteins that changed in both groups were related to non-specific inflammation. In the saliva of the septic animals there were some specific pathways activated, such as the organonitrogen compound metabolic process and lipid transport, whereas, in the serum, one of the main activated pathways was the regulation of protein secretion. Overall, saliva and serum showed different proteome variations in response to septic inflammation and could provide complementary information about the pathophysiological mechanisms occurring in this condition. Additionally, salivary aldolase A could be a potential biomarker of sepsis in pigs that should be confirmed in a larger population.

## 1. Introduction

Sepsis is defined as a systemic inflammatory response syndrome (SIRS) triggered by an infectious agent [1]. It has been recognized as a global priority by the World Health Organization and the World Health Assembly [2], as it is one of the major causes of severe illness in humans and animals [3,4]. The most challenging aspect of sepsis is its diagnosis and distinction from SIRS, which does not always involve infection but also non-infectious conditions, like trauma [5]. The misdiagnosis of a non-septic inflammation as sepsis results in a non-rational use of antibiotics, which enhances the development of multidrug-resistant bacteria leading to a “One Health” problem [6,7]. The study of the protein variations that can occur in saliva and serum in sepsis could contribute to a better knowledge of the pathophysiology of sepsis and also aid in the discovery of new potential biomarkers for this condition. This knowledge is of importance in the context of the reduction of antibiotic resistances [8], because could lead to a more accurate identification of this condition and, therefore, the adequate and reasonable use of antibiotics, which contributes to the reduction of antibiotic resistance [9,10]. This is particularly important in the pig, as it is the domestic species in which antibiotics are most used [11].

The administration of lipopolysaccharide (LPS) from Gram-negative pathogens is a recognized experimental model of sepsis induction in the pig [12]. In addition, non-septic inflammation can be induced in this species with the experimental injection of turpentine oil [13]. The usefulness of animal models in the study of sepsis has been controversial, based on the reasoning that these models do not mimic human clinical sepsis in which a hyperdynamic state occurs, in contrast to the decreased cardiac output and increased systemic vascular resistance that happens after LPS administration in animal models [14]. Another cause for concern is the balance between the benefits obtained from the study of the pathophysiology of sepsis and the well-being of the animals implicated in these experimental models [15]. However, LPS administration is considered an adequate model in pigs to study the pathophysiology of inflammation, immune system [13,16,17], and changes in biomarkers of oxidative status [18] associated with sepsis.

Analyses of saliva samples are gaining growing importance in pigs as a sample to evaluate health status since they can be collected by non-invasive procedures and with less stress compared to venipuncture [19]. In the previous studies about the inflammatory response triggered by the repeated administration of LPS [20] or the local application of turpentine [21], acute-phase proteins (e.g., C-reactive protein and haptoglobin) and stress indicators (e.g., cortisol) were reliably detected in saliva samples.

The application of proteomics allows the study of a complete protein profile of a sample, evidencing the specific alterations associated with specific metabolic pathways [22]. Gel-based proteomics has been already used to investigate the serum proteome of swine after LPS administration [23]. Nevertheless, the gel-free mass spectrometry-based proteomics has the advantage of being more sensitive and allows the high-throughput profiling of proteins providing higher quantification accuracy than the gel-based techniques [24]. The use of isobaric tagging with tandem mass tags (TMT) allows the simultaneous relative quantification of differentially labeled peptides, increasing the sensitivity of the gel-free proteomics analysis [25].

To the best of authors’ knowledge, there are no studies about proteomic in porcine saliva in pigs with sepsis. The objective of this report was to study the sepsis-related changes in the proteome of saliva and serum in the pig, with the ultimate goal of broadening the understanding of the pathophysiological mechanisms involved in sepsis and detecting possible potential biomarkers. For this purpose, we investigated the dynamic changes of proteins in saliva and serum in pigs in a model of septic inflammation induced by the administration of LPS. In addition, we used a model of non-septic inflammation through the administration of turpentine oil for comparative purposes.

## 2. Results

### 2.1. Proteomic Changes in LPS-Challenged Pigs

A total of 18 proteins showed a different relative abundance in the saliva of pigs after LPS administration (Table 1). Among these, the proteins with the highest change were fructose-biphosphate aldolase (ALDOA), serpin domain-containing protein (SERPINB12), annexin (ANXA2), moesin (MSN), and the immunoglobulins M (IgM) and G (IgG), all being upregulated. The higher magnitude of increase of these proteins was observed at 6 h post-LPS administration.

**Figure 1:**
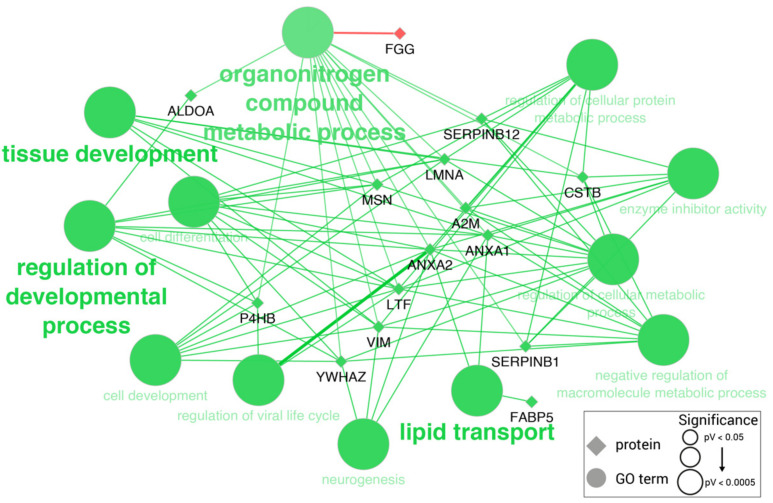
Significantly enriched GO terms among differentially expressed proteins in the saliva of pigs with sepsis. Edges link proteins (diamonds) to their associated GO terms (circles). Proteins are colored in green if overexpressed or in red if down-expressed. GO terms are colored accordingly to the proportion of over-/down-expressed proteins. GO term shape and font size is proportional to GO term significance, but all included ones showed a *p*-value < 0.05.

GO enrichment analysis indicated that the differentially expressed salivary proteins in LPS-challenged pigs were significantly associated with 11 different GO terms (Supplementary Table S1), namely, the organonitrogen compound metabolic process (GO:1901564), tissue development (GO:0009888), the regulation of the developmental process (GO:0050793), and lipid transport (GO:0006869) (Figure 1).

The serum protein profile of the LPS-challenged pigs showed significant differences in the abundances of 30 proteins after LPS administration (Table 2). Among those with the higher relative abundance, the most protruding changes at 24 h were present in two SERPIN domain-containing proteins (LOC106504547 and LOC396684), followed by haptoglobin (HP), pentraxin/C-reactive protein (CRP) and apolipoprotein (APOE). At 24 h, the proteins with lowest relative abundance were thrombospondin 1 (THBS1), vitronectin (VTN), plasma retinol-binding protein (RBP4), carboxypeptidase B2 (CPB2), and the apolipoprotein A-1 (APOA1). Although these proteins showed reduced abundance levels at 6 h post-LPS administration, they reached their lowest levels at 24 h.

**Figure 2:**
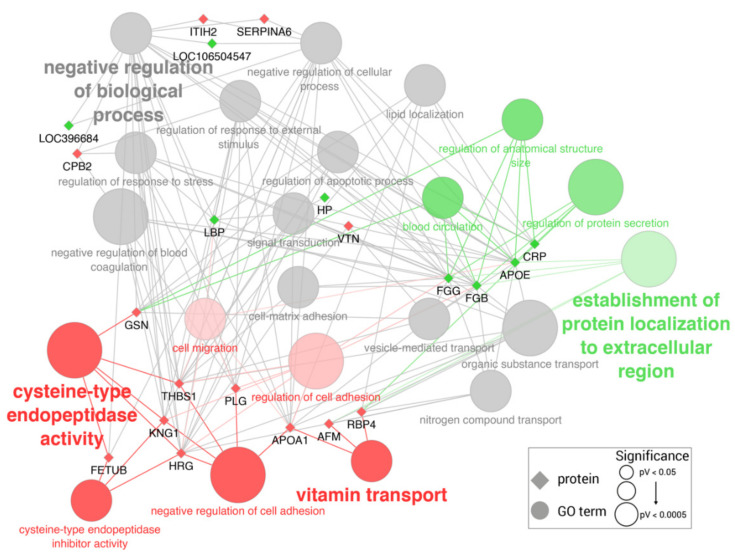
Significantly enriched GO terms among differentially expressed proteins in the serum of pigs with sepsis. Edges link proteins (diamonds) to their associated GO terms (circles). Proteins are colored in green if overexpressed or in red if down-expressed. GO terms are colored accordingly to the proportion of over-/down-expressed proteins. GO term shape and font size is proportional to GO term significance, but all included ones showed a *p* value < 0.05.

GO analysis enriched 34 GO terms among the altered serum proteins (full list in Supplementary Table S2). Downregulated proteins were more associated with cysteine-type endopeptidase activity (GO:0004197), vitamin transport (GO:0051180) or negative regulation of cell adhesion (GO:0007162), while upregulated proteins were more associated with establishment of localization to extracellular region (GO:0035592) and the regulation of protein secretion (GO:0050708). Moreover, in general, both types of proteins were functionally related to the upregulation of different biological processes, in particular, the negative regulation of biological processes (GO:0048519), the negative regulation of blood coagulation (GO:0030195), as well as the regulation of the response to external stimulus (GO:0032101) and the regulation of the apoptotic process (GO:0042981) among others (Figure 2).

### 2.2. Proteomic Changes in Turpentine-Challenged Pigs

A total of nine salivary proteins showed significantly increased abundance in pigs after turpentine administration (Table 3), while no protein showed significant decreases. The most significant increases were observed in albumin (ALB), a H4 histone (UniProtKB: P51524), and a cystatin domain-containing protein (HRG), among others. The higher upregulation levels were detected at 6 h after turpentine administration.

**Figure 3:**
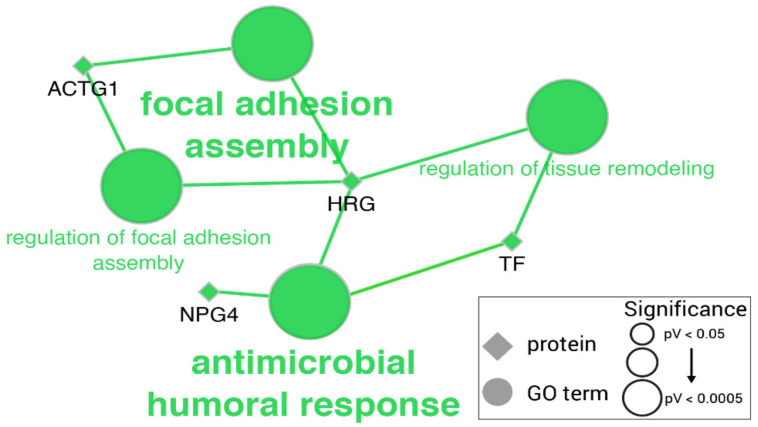
Significantly enriched GO terms among differentially expressed proteins in the saliva of pigs with non-septic inflammation. Edges link proteins (diamonds) to their associated GO terms (circles). Proteins colored in green are overexpressed. GO terms are colored accordingly to the proportion of over-/down-expressed proteins. GO term shape and font size is proportional to GO term significance, but all included ones showed a *p* value < 0.05.

GO enrichment analysis showed significant over-representation of the GO terms: antimicrobial humoral response (GO:0019730), focal adhesion assembly (GO:0048041), and serine-type endopeptidase activity (GO:0004252) (Figure 3) (full list in Supplementary Table S3).

In serum, a change in the relative abundance after turpentine administration was noted for 26 proteins (Table 4). The most upregulated proteins were CRP, two SERPIN domain-containing proteins (LOC106504547 and LOC100156325), HP, fibrinogen alpha chain (FGA), and the lipopolysaccharide-binding protein (LBP), showing their higher expressions levels 24 h post-turpentine administration. On the other hand, the most downregulated proteins overall after 24 h were APOA1, MACPF domain-containing protein (C8A), SERPINA6, plasma retinol (RBP4), and vitronectin (VTN).

**Figure 4:**
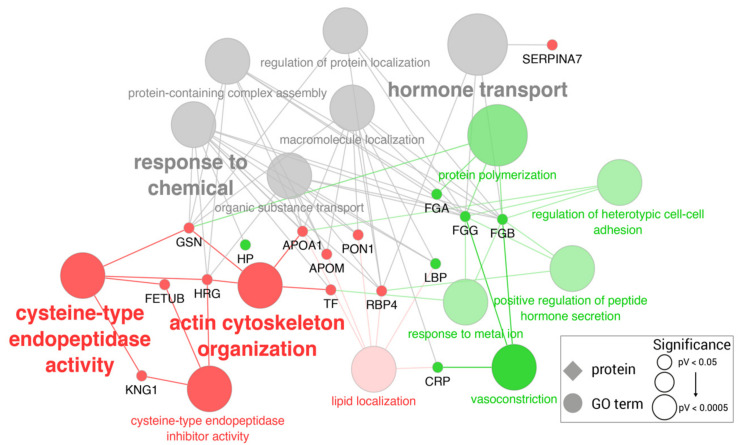
Significantly enriched GO terms among differentially expressed proteins in the serum of pigs with non-septic inflammation. Edges link proteins (diamonds) to their associated GO terms (circles). Proteins are colored in green if overexpressed or in red if down-expressed. GO terms are colored accordingly to the proportion of over-/down-expressed proteins. GO term shape and font size is proportional to GO term significance, but all included ones showed a *p* value < 0.05.

GO enrichment analysis indicated that downregulated proteins have functions related to cysteine-type endopeptidase activity (GO:0004197) and actin cytoskeleton organization (GO:0030036), while upregulated proteins participate in vasoconstriction (GO:0042310), protein polymerization (GO:0051258), and the positive regulation of peptide hormone secretion (GO:0090277). Both are equally associated with hormone transport (GO:0009914) and response to chemicals (GO:0042221) (Figure 4) (full list in Supplementary Table S4).

### 2.3. Measurement of Aldolase Activity in Porcine Saliva

The analytical validation of the ALDOA automated assay showed an intra- and inter-assay imprecision less than 10% and a high linearity (R > 0.99) after the serial dilutions of a saliva sample with high ALDOA activity. The lower limit of quantification (LLOQ) and the limit of detection (LoD) were set at 1.3 and 0.1 U/L, respectively.

**Figure 5:**
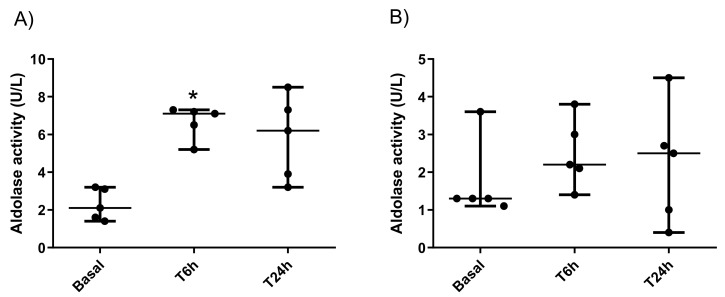
Salivary aldolase activity levels (U/L) during experimentally-induced sepsis in a model of LPS-challenged pigs (**A**) and non-septic inflammation in a model of turpentine-challenged pigs (**B**). Basal: 24 h before LPS- or turpentine-injection; T6 h: 6 h after LPS- or turpentine-injection; T24 h: 24 h after LPS- or turpentine-injection. Lines indicate the minimum, median, and maximum values. Asterisks indicate statistically significant differences (*p < 0.05) with basal time. Circles represent the sample values.

The activity of salivary ALDOA was significantly higher in pigs after LPS-induced sepsis at 6 h post injection (median 7.1 U/L and range 5.2–7.3 U/L) compared with basal values (median 2.1 U/L and 1.4–3.2 U/L) (P = 0.020), while at 24 h the ALDOA activity (median 6.2 U/L and range 3.2–8.5 U/L) did not show significant differences compared with basal values (Figure 5A). In the case of the turpentine-induced group, ALDOA activity at 6 h (median 2.2 U/L and range 1.4–3.8 U/L) and at 24 h (median 2.5 U/L and range 0.4–4.5 U/L) showed no significant differences in comparison with the basal values (median 1.3 U/L and range 1.1–3.6 U/L) (Figure 5B). No significant differences in ALDOA activities were observed between males and females in the two-way ANOVA analysis.

**Figure 6:**
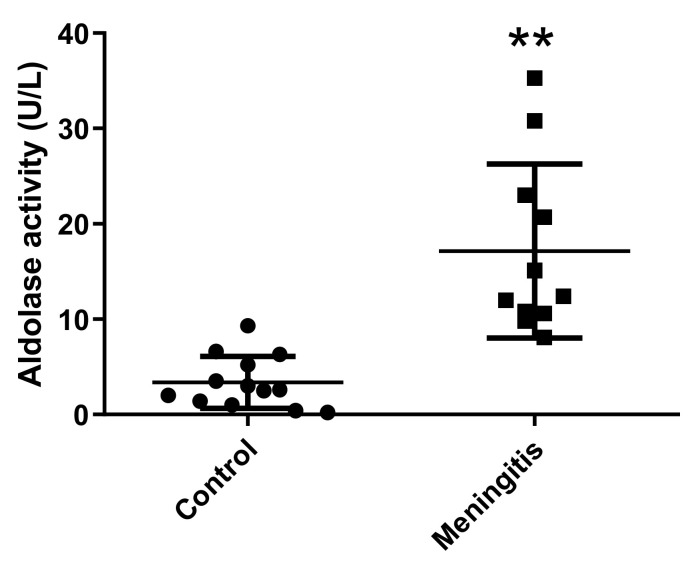
Salivary aldolase activity (U/L) in pigs with meningitis compared with healthy controls. Lines indicate the minimum, median, and maximum values. Asterisks indicate statistically significant differences (** *p* = 0.001). Circles and squares represent the sample values of control and meningitis groups, respectively.

In addition, pigs with meningitis showed significantly higher activity levels of salivary ALDOA (median 10.20 U/L and range 8.1–15.1 U/L) compared with healthy controls (median 2.80 and range 0.2–9.8 U/L) (*p* = 0.001) (Figure 6).

## 3. Discussion

In this study, we reported the changes in salivary and serum protein profiles of pigs with septic inflammation experimentally-induced by LPS administration and their pathophysiological implications. We observed a higher number of proteins changed in abundance in the saliva of the sepsis-induced group compared with the non-septic inflammation group (18 vs. 9). In addition, some of the proteins that changed in the pigs with sepsis did not have significant changes in non-septic conditions. For example, in sepsis, ALDOA, and serpin B12, which were the proteins that showed higher increases, did not display significant changes in the turpentine model.

ALDOA is a glycolytic enzyme catalyzing the reversible cleavage of fructose-1, 6-bisphosphate (Fru-1, 6-P2) into glyceraldehyde-3-phosphate (G3P) and dihydroxyacetone phosphate (DHAP). The family of ALDOA, in addition to be integrated by glycolytic enzymes, has a close relationship with muscle damage, the development of the brain, and ATP production. ALDOA increases its expression in the muscles of rats treated with LPS [26,27] and is present in the membrane surface of different pathogens where it acts as an adhesin [28,29]. Therefore, ALDOA production is directly related to the presence of sepsis by acting as a promoter of bacterial adhesion to the host cell receptors to facilitate invasion [30], thus being a potent stimulator of immune response in humans [31].

When ALDOA was measured by an automated spectrophotometric assay, it showed significant increases in pigs after the LPS administration at 6 h post-injection, as occurred in the proteomic study. In addition, our validation study found that ALDOA was higher in pigs naturally affected by meningoencephalitis caused by S. suis compared with healthy pigs. This result could reinforce the possibility of considering the salivary ALDOA as a possible biomarker of sepsis in field conditions. Gender had no influence on the ALDOA activity in the validation study. However, these results should be taken with caution and should be verified in a large population of pigs and also in pigs with sepsis produced by different infectious agents.

Serpin B12 is an inhibitor of trypsin-like serine proteinases as trypsin and plasmin [32]. The presence of this protein in epithelia and tissues suggests a role in host defense, either by the inhibition of exogenous viral and bacterial proteases or by a cytoprotective role of vital cells from endogenous proteases needed to combat infection [33]. Therefore, it could be related to the defense and compensatory effects of the organism against bacterial infection.

In this study, we also identified a set of proteins in which abundance was significantly changed in the saliva of pigs with non-septic inflammation induced by turpentine. Two specific proteins were upregulated in non-septic inflammation but showed no significant variations in the saliva of pigs with sepsis: ALB and histone H4. ALB was detected in human saliva as a part of plasma derivates or the production of salivary glands [34]. We hypothesized that the presence of albumin in the saliva of pigs could be more related to the local production of this protein than to the ultrafiltration of serum since the pigs subjected to non-septic inflammation did not show elevated levels of albumin in the serum. Further studies should be performed to clarify the reason for the increase in salivary albumin in non-septic inflammation in pigs and evaluate its use as a possible marker of this condition.

We also detected increased abundances of histone H4 in non-septic inflammation. Histones are basic proteins located in the nucleus. It is believed that histones play proinflammatory functions upon their release from the nucleus into the extracellular environment. Previous studies [35] reported their elevated levels in sepsis causing cellular injury, which creates an additional research area because our results include the elevations only in the turpentine model.

The differences found in GO analysis in the saliva of pigs with sepsis were associated with the lipid transport, tissue development, and organonitrogen compound metabolic process. Previous reports have indicated the involvement of lipids in the pathogenesis of sepsis, with lipid moieties playing a role in pathogen toxin clearance and in modulating inflammatory responses [36]. On the other hand, changes in humoral immune response and serine-type endopeptidase activity appeared in non-septic inflammation.

In the serum of both LPS and turpentine groups, the proteomic analysis showed that pigs experienced an increase in HAPT and CRP at 6 h post-stimulus. However, these proteins were not detected in saliva by proteomics, and this could be due to the low concentrations of these proteins in this fluid. Our results in serum are in line with a previous report, in which the serum proteome of swine after LPS administration was analyzed [23]. The authors indicated the upregulation of HAPT and CRP after 6 h post LPS injection. These results were further confirmed by ELISA assays and are in agreement with other studies which showed that serum concentrations of CRP and HAPT can rapidly increase during the first 4–5 h after exposure to a single stimulus [37,38]. One protein that showed differences in serum abundance between LPS- and turpentine-challenged pigs was apolipoprotein E (APOE), which showed increases in the group of LPS. The increase of this protein has been related to the risk of sepsis in human beings [39].

Serum and saliva showed different responses in our experimental conditions, with differences in the number of proteins showing significant changes as well as in the pattern of proteins with variations. These discrepancies in the number and types of proteins that change between these fluids have been observed previously in other species and diseases [40,41,42,43]. Therefore, saliva and serum could present complementary information when analyzed.

This study has some limitations that must be considered. First, we employed a small number of animals, and therefore this study should be considered as a pilot and additional studies with a larger population of animals and with different septic diseases should be carried out to confirm our findings. Additionally, gender had no influence on the ALDOA activity in the validation study, but these results should be taken with caution and should be verified in a large population of pigs and also in pigs with sepsis produced by different infectious agents. In addition, we used the basal times of each group as a reference for ideal health status but, ideally, a control group of pigs with no treatment might be included. In order to avoid the stress of a blood extraction to the pigs, only saliva was used in the validation study and, ideally, the proteomic results should also be validated in the serum of pigs with meningitis. Furthermore, the changes in the proteome of pigs with sepsis and non-septic inflammation might be accompanied by changes in the transcriptome that have not been assessed in this study and would be of interest for further research.

## 4. Materials and Methods

### 4.1. Animals

#### 4.1.1. Proteomic Study

Samples from growing male pigs (*Sus scrofa domesticus*) (Large White) in the mid fattening period from the Experimental Farm of the University of Murcia (Murcia, Spain) were used in this study (full information of animals can be found in Supplementary Table S5). The experimental procedure used to obtain these samples was described in a previous report [44]. The samples were from the following two groups:

- Lipopolisacharide (LPS) group (*n* = 5). Pigs were individually administered LPS from Escherichia coli (Sigma-Aldrich, St. Louis, MO, USA) reconstituted in sterile saline solution in a single dose of 30 ug/kg by intramuscular route as previously reported [45].
- Turpentine group (*n* = 5). Each pig was administered a total of 8 mL subcutaneous injections of turpentine oil (oil of turpentine purified, Sigma–Aldrich, St. Louis, MO, USA), 4 mL in each front flank, as previously described [46].

In all animals, LPS and the turpentine oil were administered between 8 am and 9 am. The saliva and blood samples from three collection times were analyzed in the proteomic study: 24 h before the experiment (basal) and 6 h (T6) and 24 h (T24) post-administration.

#### 4.1.2. Validation Study

For the validation study, we analyzed the changes in the saliva level of the proteins selected after the proteomic study in two different situations:

1. Septic and non-septic experimentally-induced inflammation: An aliquot of each saliva sample of the LPS and turpentine groups used in the proteomic study was analyzed.
2. Sepsis in field conditions: Two groups of Large White weaning pigs from 6 to 9 weeks old were selected from a commercial farm located in the same geographical area. One was a group of pigs diagnosed with meningitis (*n* = 11, six males and five females), and the other were clinically healthy pigs (*n* = 13, seven males and six females). The animals with meningitis had clinical signs compatible with this disease (ataxia, anorexia, lateral recumbency, and padding) [47] and were positive for the presence of Streptococcus suis in bacteriological cultures performed in blood agar plates following standard procedures [48]. Only saliva was obtained in this trial, aiming to avoid the stress associated with blood extraction.

The study protocol was approved by the Bioethical Commission of the University of Murcia, according to the European Council Directives regarding the protection of animals used for experimental purposes (CEEA 563/2019).

### 4.2. Sample Collection

Saliva was collected using a sponge clipped to a flexible thin metal rod of approximately 10 cm in length. Pigs were allowed to chew on the sponge until it was thoroughly moist. Then the sponges were removed from the pigs’ mouths and placed in Salivette tubes (Sarstedt, Aktiengesellschaft & Co., D-51588 Nümbrecht, Germany). In all sampled pigs, saliva was first collected, and, after that, the animals were restrained with a nose sling to obtain a blood sample by the venipuncture of the jugular vein using vacuum plain tubes (BD Vacutainer, Franklin Lakes, NJ, USA). All samples were kept at 4−8 °C until arrival at the laboratory, where the vacutainer and the Salivette tubes were centrifuged at 3000× *g* and 4 °C for 10 min to obtain serum and saliva supernatant, respectively. Then, the aliquots were transferred into the Eppendorf tubes and stored at −80 ºC until the analysis was performed.

### 4.3. Sample Preparation for Proteomic Analysis

Proteomic analysis of saliva and serum samples was performed by TMT-based quantitative approach as described previously [49].

Briefly, saliva samples were centrifuged (13,000× *g*, 10 min, 4 °C), precipitated overnight in the ice-cold acetone, the supernatant resuspended in 1% SDS in 0.1 M triethyl ammonium bicarbonate (TEAB, Thermo Scientific, Rockford, IL, USA) buffer, and protein concentration was determined using the BCA assay (Thermo Scientific, Rockford, IL, USA). Furthermore, the preparation protocol was identical for the serum and saliva samples. An amount of 35 µg from the samples and internal standards (a pool of equal protein amounts from all samples) was reduced with dithiothreitol (DTT) (Sigma-Aldrich, St. Louis, MO, USA), alkylated with iodoacetamide (Sigma-Aldrich, St. Louis, MO, USA), and precipitated overnight with ice-cold acetone (VWR, Radnor, PA, USA). Following the centrifugation (9000× *g*, 4 °C, 15 min), the protein pellets were dissolved in 0.1 M TEAB and digested using 1 mg/mL trypsin (Trypsin Gold, Promega; trypsin-to-protein ratio 1:35, at 37 °C overnight). TMT 6plex reagents (Thermo Scientific, Rockford, IL, USA) were prepared according to the manufacturer’s procedure. An amount of 19 μL of the specific TMT label was added to each sample. After 60 min at the room temperature the addition of 5% hydroxylamine (Sigma-Aldrich, St. Louis, MO, USA) quenched the reaction. Five TMT-modified samples were randomly combined with the internal standard, aliquoted, dried, and then liquid chromatography–tandem mass spectrometry (LC-MS/MS) analysis proceeded.

### 4.4. Liquid Chromatography-Tandem Mass Spectrometry (LC-MS/MS) Analysis

For LC-MS/MS analysis, we used the platform consisting of the Ultimate 3000 RSLCnano system (Dionex, Germering, Germany) and the Q Exactive Plus mass spectrometer (Thermo Fisher Scientific, Bremen, Germany). Following their dissolution in the loading solvent (2% ACN, 0.1% formic acid), the labelled peptides were loaded onto the trap column (C18 PepMap100, 5 μm, 100A, 300 μm × 5 mm). After that, the separation on the analytical column (PepMap™ RSLC C18, 50 cm × 75 μm) followed. For achieving the separation gradient, we used two mobile phases, one was 0.1% formic acid in water (mobile phase A) and the other was 0.1% formic acid in 80% acetonitrile (mobile phase B). The separation protocol involved the linear gradient of 5–55% mobile phase B over 120 min, followed by 55% to 95% for 1 min, 95% for 2 min, and a decrease to 5% B during 20 min under the flow rate of 300 nL/min. The nanospray Flex ion source (Thermo Fisher Scientific, Bremen, Germany) with the 10 μm inner diameter SilicaTip emitter (New Objective, Littleton, MA, USA) was used for ionization. The MS operating parameters were a positive ion mode using DDA Top8 method, full-scan in range from *m*/*z* 350.0 to *m*/*z* 1800.0, resolution 70,000, 120 ms injection time, AGC target 1 × 106, isolation window ±2.0 Da, and the dynamic exclusion 30 s. The conditions for HCD fragmentation were step collision energy (29% and 35% NCE) with a resolution of 17,500 and AGC target of 2 × 105. The criteria to exclude the precursor ions from fragmentation were the unassigned charge state or the charge states +1 and higher than +7.

For protein identification and quantification, we followed the SEQUEST algorithm with the Proteome Discoverer software (version 2.3., ThermoFisher Scientific, Bremen, Germany), searching against Sus scrofa FASTA files (downloaded from Uniprot database on December 2, 2020, 150,392 sequences). The identification parameters were: two trypsin missed cleavage sites, precursor and fragment mass tolerances of 10 ppm and 0.02 Da, respectively; carbamidomethyl (C) fixed peptide modification; oxidation (M); and TMT sixplex (K, peptide N-terminus) dynamic modifications. The false discovery rate (FDR) was set at 5%, as calculated with the Percolator algorithm in the Proteome Discoverer workflow. Only the confidently identified proteins (at least two unique peptides and 5% FDR) entered the bioinformatics analysis.

### 4.5. Bioinformatics

For Fold changes between groups were calculated as the log2(Mean(Group2)/Mean(Group1)). To test the significance of the difference in the relative abundance between the samples taken from the same group at the different time points, we applied the Friedman test with Dunn’s multiple comparison test as the post-hoc analysis. Statistical significance was considered when *p* < 0.05. All statistical analyses were implemented using Python3 and the SciPy (Virtanen et al., 2020) and scikit-post hoc libraries.

Proteins were mapped to UniProt entries and annotated with the gene names, protein names, and descriptions. For the functional characterization of the differentially expressed proteins, Gene Ontology (GO) enrichment analysis was performed using the Cytoscape plugin ClueGo and its functionalities in order to fuse and group functionally related terms to reduce redundancy and REVIGO [50].

### 4.6. Validation Study

ALDOA was the protein selected for the validation study. The first step was the validation of the commercially available reagent kit (Aldolase, Randox Laboratories Ltd., Crumlin, UK) for the measurement ALDOA activity in saliva. This assay was applied to Beckman Coulter AU 400 autoanalyzer following the manufacturer’s recommendations.

The validation was based on the following four features:

- Precision: The intra- and inter-assay coefficient of variation (CV) were calculated after analyzing two saliva samples of high and low concentration, respectively.
- Accuracy: The indirect evaluation by the linearity under the dilution of a saliva sample with a high ALDOA level.
- LLOQ: The lowest analyte concentration that could be measured with an intra-assay CV < 20%.
- LD: The lowest analyte concentration that could be distinguished from zero value. It was calculated based on data from ten replicate measurements of the zero standard (saline solution) as a mean value plus three standard deviations (SD).

Once validated, the assay was used to measure ALDOA in the saliva of LPS and turpentine pigs used in the proteomic study and for the comparison between pigs with meningitis and healthy pigs, as was indicated previously. The Friedman test followed by Dunn’s multiple comparisons post hoc test was used to assess the significance between the three time points (basal, T6 h, and T24 h). In the case of pigs with meningitis and controls, the Mann–Whitney U test was used to compare salivary ALDOA activity. Additionally, the influence of gender in ALDOA measurements was investigated by a two-way ANOVA test. Results were expressed as median, and ranges and the difference were considered significant when the *p*-value was below 0.05.

## 5. Conclusions

The saliva and serum proteome of pigs showed changes in sepsis and also in non-septic inflammation in our experimental conditions. Aldolase A and serpin 12 were proteins in saliva that were significantly upregulated in sepsis and did not show significant changes in non-septic inflammation. In addition, GO analysis showed that different pathophysiological pathways in saliva were altered in pigs with sepsis compared with pigs with non-septic inflammation. In serum, increases in acute phase proteins were detected in both conditions and some proteins, such as APOE, showed significant changes in sepsis but not in non-septic inflammation.

Overall, these results indicate that proteins in saliva and serum can change, reflecting different pathophysiological mechanisms in sepsis and non-septic inflammation and some of these proteins could be potential biomarkers for these conditions. This should be considered as a pilot study since it included only male pigs in the proteomic study and the results were validated only in meningitis as a sepsis-related pathology but not in other infections.

## Figures and Tables

**Table 1:** Differentially expressed salivary proteins in pigs with experimentally-induced sepsis.

		Mean Abundances			Fold Changes	
Gene (or Accession Number)	Protein Name	Basal	6 h	24 h	6 h/Basal	24 h/Basal
ALDOA	Fructose-biphosphate aldolase	0.50	1.13	0.88	1.18 **	0.83
SERPINB12	SERPIN domain-containing protein	0.42	0.92	0.74	1.12 *	0.80
ANXA2	Annexin	0.50	1.02	0.65	1.04 *	0.39
SFN	14-3-3 sigma protein	0.61	1.23	0.94	1.02 **	0.63
MSN	Moesin	0.67	1.34	1.12	0.99 *	0.74
SERPINB1	Leukocyte elastase inhibitor	0.74	1.35	0.97	0.88 *	0.39
IGHA	IgM	0.64	1.16	0.97	0.86 **	0.59
ECH1	Galectin	0.61	1.07	0.93	0.81 *	0.60
FABP5	FABP domain-containing protein	0.66	1.06	1.16	0.70	0.82 *
A2M	Alpha-2-macroglobulin isoform a	0.80	1.28	1.01	0.68 **	0.33
IGHG	IgG heavy chain	0.82	1.28	0.80	0.63 *	-0.04
LMNA	Lamin isoform A	0.74	1.11	1.01	0.59 *	0.45
P4HB	Protein disulfide-isomerase	0.79	1.19	1.06	0.59 *	0.43
TKT	Transketolase	0.75	1.13	1.03	0.59 *	0.45
YWHAZ	14-3-3 protein zeta/delta	0.64	0.86	1.13	0.43	0.83 *
CSTB	Cystatin-B	0.80	1.06	1.35	0.40	0.75 *
LCN2	Neutrophil gelatinase-associated lipocalin	0.72	0.81	1.31	0.18	0.86 *
P51524 (accession)	Prophenin and tritrpticin precursor (Fragment)	0.73	0.54	1.71	−0.43	1.23 *

* *p*-value < 0.05; ** *p*-value < 0.01.

**Table 2:** Differentially expressed serum proteins in pigs with experimentally-induced sepsis.

		Mean Abundances			Fold Changes	
Gene (or Accession Number)	Protein Name	Basal	6 h	24 h	6 h/Basal	24 h/Basal
LOC106504547	SERPIN domain-containing protein	0.78	1.02	1.66	0.43	1.13 ***
LOC396684	SERPIN domain-containing protein	0.90	1.07	1.51	0.25	0.74 **
HP	Haptoglobin	0.77	0.98	1.21	0.34	0.63 **
CRP	Pentaxin/C-reactive protein	0.56	1.09	0.86	0.96 **	0.62
APOE	Apolipoprotein E	0.97	1.14	1.04	0.23 *	0.10
LBP	Lipopolysaccharide-binding protein	0.84	0.95	1.08	0.17	0.36 *
LUM	Lumican	0.94	0.99	1.18	0.07	0.32 *
A0A480XY00	Complement C1s subcomponent isoform 1 preproprotein	0.98	0.98	1.10	−0.01	0.16 **
FGB	Fibrinogen beta chain	0.94	0.78	1.10	−0.26 **	0.22
FGG	Fibrinogen C-terminal domain-containing	0.99	0.81	1.06	−0.29 *	0.10
FGA	Fibrinogen alpha chain	0.96	0.78	1.02	−0.29 *	0.08
FN1	Fibronectin	1.03	0.91	0.95	−018 *	−0.12
ALB	Albumin	1.01	1.00	0.93	−0.02	−0.12 *
SERPINC1	Antithrombin-III	0.99	0.94	0.90	−0.06	−0.13 *
SERPINA7	Thyroxine-binding globulin	1.02	0.99	0.92	−0.10	−0.14 *
ITIH1	Inter-alpha-trypsin inhibitor heavy chain H1 isoform a preproprotein	1.06	1.00	0.95	−0.08	−0.14 *
C8B	Complement component 8 subunit beta	1.01	1.04	0.90	0.04	−016 *
PROC	Vitamin K-dependent protein C	1.16	1.08	1.02	−010	−0.19 *
ITIH2	Inter-alpha-trypsin inhibitor heavy chain H2	1.07	1.02	0.92	−0.06	−0.22 *
A0A4X1TBX0	C1q domain-containing protein	1.01	0.95	0.86	−0.08	−0.22 *
C8G	Complement component C8G	1.03	1.04	0.88	0.01	−0.23 *
AFM	Afamin	1.09	1.03	0.92	−0.07	−0.24 *
SERPINA6	SERPIN domain-contaning protein	1.03	1.03	0.85	−0.01	−0.27 *
PLG	Plasminogen	1.02	1.00	0.84	−0.02	−0.27 **
GSN	Actin-depolymerizing factor	1.06	1.05	0.87	−0.01	−0.27 *
FETUB	Fetuin-B isoform 1	1.11	0.99	0.01	−0.17	−0.28 **
HRG	Histidine-rich glycoprotein	1.00	0.97	0.80	−0.03	−0.31 **
APOA1	Apolipoprotein A-1	1.13	1.02	0.90	−0.15	−0.32 *
CPB2	Carboxypeptidase B2 isoform 1 preproprotein	1.06	1.06	0.84	-0.01	−0.32 **
VTN	Vitronectin	1.09	0.92	0.82	−0.24	−0.40 **

* *p*-value < 0.05; ** *p*-value < 0.01; *** *p*-value < 0.001.

**Table 3:** Differentially expressed salivary proteins in pigs with experimentally-induced non-septic inflammation.

		Mean Abundances			Fold Changes	
Gene	Protein Name	Basal	6 h	24 h	6 h/Basal	24 h/Basal
P62802	Histone H4	0.49	1.13	0.73	1.18 *	0.55
ALB	Albumin	0.54	1.12	0.76	1.05 **	0.50
HRG	Cystatin domain-containing protein	0.67	1.25	0.90	0.88 *	0.42
A2M	Alpha-2-macroglobulin isoform a	0.61	1.03	0.73	0.75 *	0.24
TF	Beta-1 metal-binding globulin	0.72	1.14	0.93	0.65 **	0.37
IGHG	IgG heavy chain	0.67	1.05	0.90	0.64 *	0.42
P51524 (accession)	Prophenin and tritrpticin precursor (Fragment)	0.54	0.82	1.46	0.58	1.41 *
LOC106504547	SERPIN domain-containing protein	0.69	0.90	1.15	0.37	0.73 **
LCN2	Neutrophil gelatinase-associated lipocalin	0.70	0.89	1.17	0.34	0.72 *

* *p*-value < 0.05; ** *p*-value < 0.01.

**Table 4:** Differentially expressed serum proteins in pigs with experimentally-induced non-septic inflammation.

		Mean Abundances			Fold Changes	
Gene (or Accession Number)	Protein Name	Basal	6 h	24 h	6 h/Basal	24 h/Basal
CRP	Pentaxin or C-reactive protein	0.46	0.66	1.30	0.50	1.47 *
LOC106504547	SERPIN domain-containing protein	0.688	0.73	1.74	0.08	1.32 **
LOC100156325	SERPIN domain-containing protein	0.69	0.83	1.13	0.27	0.71 *
HP	Haptoglobin	0.80	0.87	1.29	0.10	0.67 **
FGA	Fibrinogen alpha-chain	0.85	0.91	1.32	0.09	0.62 **
LBP	Lipopolysaccharide-binding protein	0.90	0.98	1.34	0.11	0.57 **
FGB	Fibrinogen beta chain	0.86	0.91	1.19	0.09	0.47 **
FGG	Fibrinogen C-terminal domain-containing protein	0.91	0.93	1.21	0.02	0.42 **
A0A4X1U9T5 (accession)	Ig-like domain-containing protein	0.97	0.95	1.11	−0.02	0.19 *
APOA1	Apolipoprotein A-1	1.25	1.13	0.72	−0.13	−0.8 **
C8A	MACPF domain-containing protein	1.27	1.11	0.90	−0.19	−0.48 *
RBP4	Plasma retinol-binding protein	1.10	1.07	0.79	−0.03	−0.47 *
SERPINA6	SERPIN domain-containing protein	1.12	1.01	0.82	−0.14	−0.45 **
VTN	Vitronectin	1.19	1.16	0.92	−0.03	−0.36 **
APON	Ovarian and testicular apolipoprotein N	1.08	0.99	0.85	−0.13	−0.34 **
ITIH1	Inter-alpha-trypsin inhibitor heavy chain H1 isoform a preproprotein	1.01	1.03	0.83	0.02	−0.29 **
HRG	Histidine-rich glycoprotein	1.09	1.04	0.89	−0.06	−0.28 *
GSN	Actin-depolymerizing factor	1.03	0.99	0.88	−0.05	−0.23 *
FETUB	Fetuin-B isoform 1	1.11	1.09	0.95	−0.02	−0.23 *
TF	Serotransferrin	1.06	1.07	0.91	0.01	−0.22 *
A0SEH3 (accession)	Complement component C8G	1.00	0.98	0.88	−0.03	−0.18 *
ITIH2	Inter-alpha-trypsin inhibitor heavy chain H2	1.05	1.03	0.92	−0.02	−0.18 *
AMBP	Alpha-1-microglobulin	1.01	0.98	0.89	−0.04	−0.18 *
PROC	Vitamin K-dependent protein	1.10	1.07	0.99	−0.04	−0.15 *
A1BG	Alpha-1B-glycoprotein	1.06	1.04	0.97	−0.01	−0.12 *
A1BG	Alpha-1B-glycoprotein	1.06	1.04	0.97	−0.01	−0.12 *

* *p*-value < 0.05; ** *p*-value < 0.01.

## Data Availability

Not applicable.

## Supplementary Materials

The following supporting information can be downloaded at: https://www.mdpi.com/article/10.3390/ijms23126738/s1.
