# Supplementary material for: A Proteomic Approach to Elucidate the Changes in Saliva and Serum Proteins of Pigs with Septic and Non-Septic Inflammation
Source: Int J Mol Sci. 2022 Jun 16;23(12):6738. doi: 10.3390/ijms23126738 (PMC9223627; doi:10.3390/ijms23126738)
Supplement: Supplementary file 1 [file ijms-23-06738-s001.zip › Supplementary Table S5.pdf]

Supplementary Table S5. Complementary information of the male Large White pigs submitted to experimentally-induced septic (LPS) and non-septic (TURP) inflammation.

Group	Pig ID	Weight (kg)	Sampling time	Rectal temperature (ºC)	WBCB ($x10^3$ cells/μL)	CRP (μg/mL)
LPS	1	46.5	Basal	39.2	17.6	5.8
			T6	41.1	19.6	42.3
			T24	38.6	19.3	79.2
	2	61.5	Basal	39.1	10.2	5.6
			T6	40.3	11.9	15.9
			T24	38.9	18.6	54.8
	3	53	Basal	39.6	15.4	14.2
			T6	41.3	7.8	80.4
			T24	38.3	37.0	78
	4	53.5	Basal	39.4	12.8	6.6
			T6	41.3	6.0	45.9
			T24	38.4	17.4	64.6
	5	49	Basal	39.3	12.5	5.9
			T6	41.9	8.2	40.5
			T24	40.4	18.6	103.8
TURP	6	46	Basal	39.2	14.0	5.5
			T6	39.9	24.0	20.9
			T24	39.8	20.0	112.1
	7	52	Basal	39.5	15.2	4.1
			T6	40.4	15.9	17.7
			T24	39.4	17.1	115.7
	8	50	Basal	39.1	13.0	4.9
			T6	39.8	9.9	12
			T24	39.3	9.1	104.7
	9	52.5	Basal	39.2	17.8	6.8
			T6	39.1	22.0	16.4
			T24	38.3	17.4	91.9
	10	48	Basal	39.4	15.6	5.2
			T6	39.9	26.4	20.1
			T24	39	19.7	112.1

LPS: lipopolysaccharide; TURP: turpentine; WBCB: white blood cell count from the basophil method; CRP: C-reactive protein.
